# Notes and News

**Published:** 1905-02-11

**Authors:** 


### NOTES AND NEWS.

THE Queen has consented to be the patroness of Mrs. Gladstone's Free Convalescent Homes for the Poor of London, founded by Mrs. Gladstone in 1866. This charity has admitted upwards of 35,000 patients, men, women, and children. Originated at Woodford in Essex, it is now at Ravensbury House, Mitcham, Surrey.

THE illness of Princess Victoria has progressed so favourably that on Saturday the medical men in attendance signified that no further bulletin would be issued. The ceremony of changing the guard at Buckingham Palace, which on the day of, and since the operation on Princess Victoria, had taken place at St. James's Palace, was resumed at Buckingham Palace on Saturday morning with full musical honours.

PRINCESS HENRY OF BATTENBERG, as President of the James Memorial Cottage Hospital, East Cowes, presided last week at the annual general meeting of the institution, when the reports as to the finances and the general work were adopted.

As the result of the visit of French physicians and surgeons to London last October, the Paris doctors have invited their British brethren to pay them a return visit in May. The invitation has been accepted on behalf of the profession by a committee of which Sir William Broadbent is chairman.

THE Pope has appointed Sir Francis Cruise, M.D., who is Honorary Physician in Ordinary to the King in Ireland, a Knight of the Order of St. Gregory the Great, in special recognition of his work in connection with the "De Imitatione" of Thomas à Kempis and its literature. Sir Francis is the author of a life of Thomas à Kempis.

THE Rev. Stephen Nottidge Tebbs, of Hillside Henbury, Gloucester, who died on December 19th has bequeathed £1,000 to Bristol Eye Hospital and, subject to certain private bequests, the residue of his estate (probably about £30,000), to the Cancer Hospital, Brompton, the Royal Hospital for Incurables, Putney, the Bristol Royal Infirmary, the Bristol General Hospital, the Bristol Dispensary, and the Society for the Propagation of the Gospel.

PLAGUE has broken out in Rangoon. Many cases have been reported and a further increase is feared. Every precaution is being taken to restrict the outbreak.

A BULLETIN issued at Potsdam on Tuesday morning states that the general condition of Prince Eitel Friedrich is so good that no further bulletins will be issued.

IT is reported that Dr. Elizabeth Aschseim, of San Francisco, from repeated exposures to the $x$-rays with which she has experimented for a number of years, has sustained burns necessitating the amputation of her right arm.

IT has been widely stated that important changes in the Naval Medical Service are about to come into operation. One of the new regulations is said to be that in future civilian doctors will be invited to take service in the fleet for a short term of years. The Director-general of the Medical Department of the Admiralty informs us, however, that nothing is known in that department with reference to the alleged regulations.

AT the Devon Assizes on Tuesday a medical man was sued for fraudulent misrepresentation in connection with the sale of a practice at Bideford and Appledore. The amount paid was £1,000, and the plaintiff, also a medical man, alleged that the defendant, by paying unnecessary visits, and by various other artifices, had fictitiously increased the value of the practice. The jury found that the practice had been built up for sale, and they awarded the plaintiff £200 damages. The moral of the case is that any intending purchaser of a practice should take the precaution of employing a skilled accountant before parting with his money.

SOME interesting returns have been issued by the chief surgeon with General Oku's army. It is stated that there have only been 40 deaths from disease in his entire command from May 6th to December 22nd. The figures show that of the 24,642 cases treated up to December 1, 18,578 recovered, 40 died, and 5,609 were sent to Japan. There were 193 cases of typhoid, 342 of dysentery, and no fewer than 5,070 of beri-beri. Sixteen per cent. of the wounded died, 19 per cent. recovered on the field, and 65 per cent. were sent to Japan. Eighty-five per cent. of the wounds were caused by rifle bullets, 8 per cent. by shells, and 7 per cent. by bayonets or sword thrusts.

ONE of the extern midwifery clerks of the London Hospital has had an unusual experience. He attended a case in Wentworth Street Buildings, and having satisfied himself that the accouchement could not occur for some time, he expressed his intention of leaving and calling again later on, whereupon he was told that he could not go. While he was trying to convince a female friend of the patient at midnight that there was no immediate need, he was imprisoned and was not allowed to leave until after the event had taken place, several hours later. Subsequently a charge of unlawful imprisonment against two persons was brought by the Public Prosecutor at his instance, and the defendants have been committed for trial.

